# Outcomes of POCUS‐Guided Peripheral Intravenous Access in Difficult Venous Access Patients: A Systematic Review and Meta‐Analysis

**DOI:** 10.1002/jcu.24059

**Published:** 2025-05-07

**Authors:** Hany A. Zaki, Hussam Elmelliti, Benny Ponappan, Ahmed Shaban, Amira Shaban, Mohammed F. Abosamak, Eman E. Shaban

**Affiliations:** ^1^ Emergency Medicine Department Hamad Medical Corporation Doha Qatar; ^2^ College of Medicine Qatar University Doha Qatar; ^3^ Internal Medicine Department Mansoura University Hospital Mansoura Egypt; ^4^ Department of Anesthesia and Intensive Care Medicine, Faculty of Medicine Tanta University Tanta Egypt; ^5^ Cardiology Department Al Jufairi Diagnosis and Treatment, MOH Qatar

**Keywords:** difficult venous access, perioheral intravenous access, POCUS

## Abstract

Point Of Care Ultrasound (POCUS) guided peripheral intravenous (PIV) access offers a safer and more efficient alternative for patients with difficult venous access. A systematic review of 15 studies with 1485 patients, sourced from five databases, including PubMed, Cochrane Library, MEDLINE, Web of Science, and Google Scholar, showed POCUS significantly improved success rates (OR: 4.25), first‐attempt success (OR: 5.31), reduced attempts (MD: −0.83), and decreased procedure time (MD: −9.75 min). Fewer complications (OR: 0.49) observed with POCUS compared to standard techniques. Statistical analyses were conducted using Review Manager, and quality was assessed with Cochrane Risk of Bias and Newcastle Ottawa tools. Findings support POCUS as a safe, time‐saving procedure for improving patient outcomes.

## Graphical abstract

POCUS‐guided peripheral intravenous (PIV) access significantly improves success rates in patients with difficult venous access. This technique leads to higher first‐attempt success, fewer attempts, shorter procedure times, and reduced complications compared to standard methods, making it a safer and more efficient option for challenging cases.

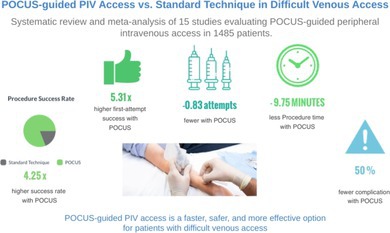

## 1 Introduction

Peripheral intravenous (PIV) access is a standard and essential clinical procedure performed in healthcare settings to administer fluids, medications, blood products, and other IV therapies (Alexandrou et al. 2018). Traditionally, this procedure is performed using the landmark approach, which requires knowledge of vascular anatomy to estimate the location of the targeted vessel and requires vessel visualization or palpation for accurate puncture (Troianos et al. 2011). However, some patients might have difficult venous access due to obesity, history of IV drug use, chronic illness, or vascular pathology (Eren 2021; van Loon et al. 2022; McKinney et al. 2023). Such patients are often subjected to multiple insertion attempts by different operators, thus increasing discomfort, lowering patient satisfaction, and delaying blood draws and laboratory test results. As a common consequence, patients with difficult PIV access undergo central venous access, which is more invasive, time‐consuming, and associated with severe complications such as arterial puncture, pneumothorax, and deep vein thrombosis (DVT) (Ouriel 2003; Au et al. 2012; Acar et al. 2009).

In recent years, point‐of‐care ultrasound (POCUS) has emerged as a valuable tool for guiding several medical procedures. For instance, our previous meta‐analysis suggested that ultrasound‐guided regional anesthesia is superior to parenteral opioids in patients presenting to the emergency department (ED) with hip fractures (A. Zaki et al. 2022). Evidence has also shown that ultrasound‐guided central venous access significantly reduces the number of puncture attempts and complications compared to the traditional landmark approach (Hind et al. 2003; Keenan 2002; Brass et al. 2015). This has led to the adoption of POCUS in guiding PIV access, specifically in patients with difficult venous access. Therefore, the current meta‐analysis will investigate the efficacy and safety of POCUS‐guided PIV access in patients with difficult venous access by synthesizing evidence from randomized and observational studies. As such, this meta‐analysis can provide valuable insights that inform clinical practice, guide future research, and ultimately improve patient care in this challenging population.

## 2 Methods

### 2.1 Information Sources and Searches

Five databases, including PubMed, Cochrane Library, MEDLINE, Web of Science, and Google Scholar, were comprehensively searched for records published from inception until October 2024. Moreover, bibliographies of records affiliated with our research were scrutinized for additional studies. The search strategy employed in each electronic database involved combining keywords such as Point‐of‐care ultrasound, peripheral intravenous access, and difficult venous access using the Boolean expressions “AND” and “OR.” The search was limited to records authored in English only. Furthermore, gray literature containing unpublished data was eliminated to minimize publication bias and improve our scientific research. The complete search strategy is detailed in the Supporting Information (Appendix A).

### 2.2 Eligibility Criteria

Two independent investigators screened full‐text records according to the following PICOST criteria. Patients (P): Adult or pediatric patients with difficult venous access. Intervention (I): POCUS‐guided peripheral intravenous catheter placement. Comparison: Standard techniques for PIV line placement. Outcomes: These included successful cannulation, procedural time, number of attempts, and adverse events. S: Randomized clinical trials (RCTs) and observational cohort studies. T: No time limitation was specified, as all records from inception until October 2024 were eligible.

Studies that did not align with the PICOST criteria and those designed as case reports, case series, editorials, conference abstracts, ongoing clinical trials, narrative reviews, and meta‐analyses were excluded. Studies evaluating POCUS‐guided peripheral intravenous catheter placement without control groups and those assessing ultrasound‐guided central intravenous access were also excluded.

### 2.3 Data Extraction and Data Items

Two independent investigators screened full‐text records of included studies and extracted data needed for a comprehensive analysis using a standardized MS Excel spreadsheet. The data collected included the name of the primary author, publication year, design of the study, study location (country), pertinent features of the enrolled participants (number of patients, study population, age, and sex), study setting, operators and their experience with ultrasound, ultrasound machine used, and the reported outcomes. All discrepancies in the extracted data were resolved via constructive discussions between the investigators or by consulting another investigator.

The primary endpoints of the present systematic review were successful PIV line placement and placement at first attempt. On the other hand, secondary outcomes were procedure time (time until successful placement of PIV line), the mean number of attempts, and complications. The complications were further subdivided into arterial puncture, hematoma, dislocation, obstruction, accidental removal, malfunctioning, nerve pain, extravasation, and ecchymosis.

### 2.4 Quality Appraisal

Study methodological quality was assessed using the Cochrane Risk of Bias (RoB) tool and Newcastle Ottawa Scale (NOS). The RoB tool was used to evaluate RCTs based on the random sequence generation, allocation concealment, blinding of participants, blinding of outcome assessment, incomplete outcome data, selective outcome reporting, and other biases. A low risk of bias, denoted by a green color, was used to indicate the absence of bias, while a high risk of bias, denoted by a red color, revealed the presence of bias. Unclear risk was not denoted by any color and was used in cases where a clear judgment on the risk of bias could not be made. On the other hand, observational studies were assessed using the NOS (Stang 2010). An NOS score of < 3 indicated poor quality, whereas scores between 4 and 6 and > 7 indicated fair and reasonable quality, respectively.

### 2.5 Data Synthesis

Statistical analyses were performed using the Review Manager software (RevMan version 5.4.1). Dichotomous data was pooled using the simple odds ratio (OR), while continuous data was calculated for the mean difference. Outcomes displaying significant interstudy heterogeneity were pooled using the random‐effects model, and those with low heterogeneity were pooled using the fixed‐effect model. The heterogeneity across the studies was measured with the *I* ^2^ statistics, of which values above 50% indicated significant heterogeneity (Higgins et al. 2002, 2003). Statistical significance in all tests was suggested by a two‐sided *p*‐value of less than 0.05. Moreover, the pooled results were presented in forest plots and were accompanied by their corresponding 95% confidence interval (CI). In studies where continuous data was presented in terms of median and range or interquartile range, the mean and standard deviations were estimated using the formula provided by Wang and colleagues (Wan et al. 2014).

We also conducted subgroup analyses to investigate the source of heterogeneity. These subgroup analyses involved dividing the studies according to the study design (RCTs and observational studies), study setting (ED, intensive care unit (ICU), and anesthesia unit), participant's age group (Adults and children), view for venous visualization (transverse and longitudinal), and technique for ultrasound guidance (dynamic and static).

## 3 Results

### 3.1 Study Selection

**FIGURE 1:**
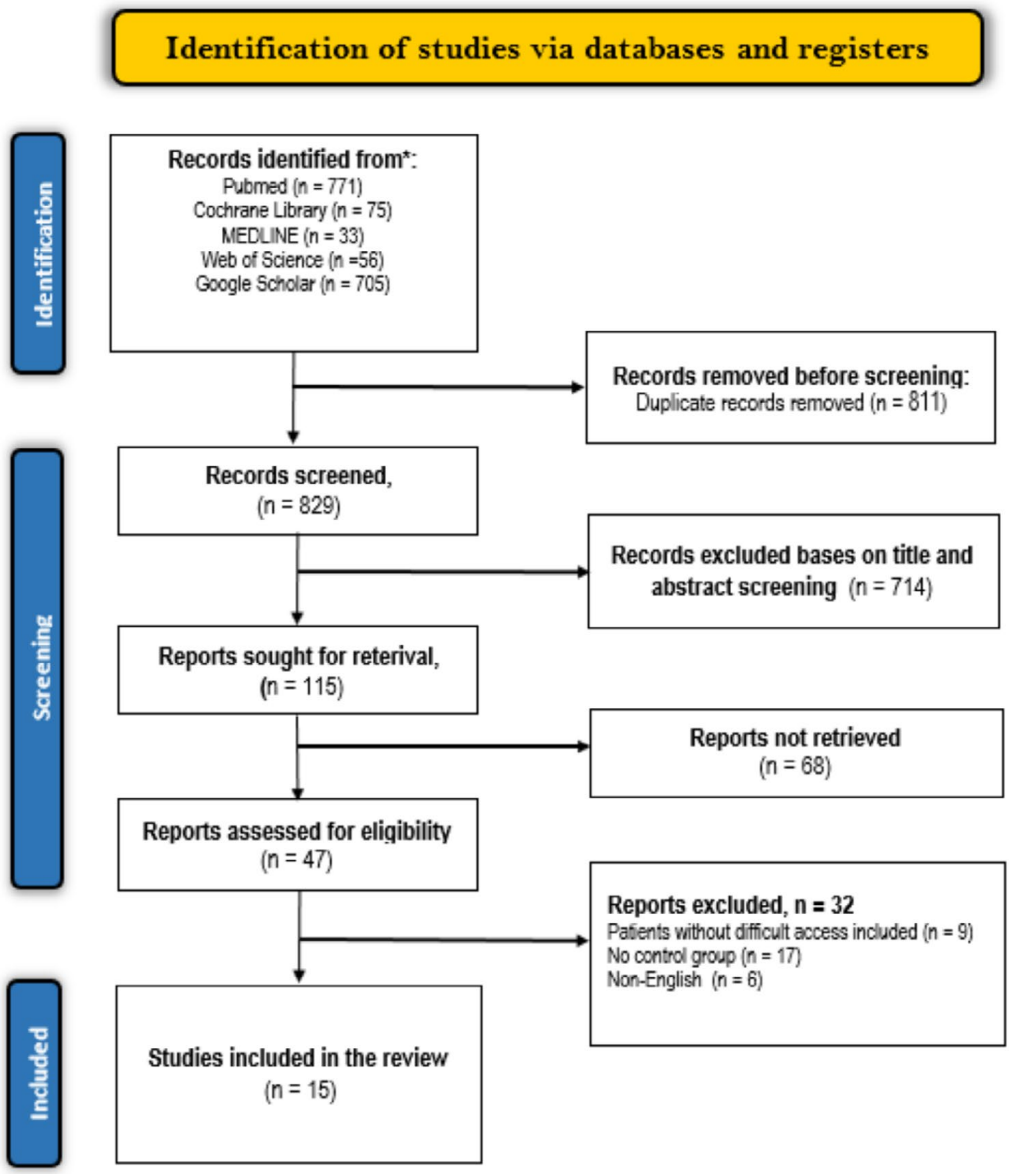
PRISMA flow diagram for study selection.

The search strategy resulted in 1640 potentially relevant studies. Out of these, 811 duplicates were eliminated. Title and abstract screening further eliminated 714 records, which were irrelevant to our research topic. Furthermore, 68 records designed as ongoing clinical trials, case reports, conference abstracts, narrative reviews, and meta‐analyses were eliminated, leaving 47 potential full‐text studies. These studies underwent screening using the predefined eligibility criteria, of which 32 were excluded for the following reasons: 6 were not published in English, 17 did not have control groups, and 9 involved patients without difficult venous access (Figure 1).

### 3.2 Characteristics of the Included Studies

Fifteen studies involving 1485 patients with difficult venous access were included in the analysis. These studies were conducted across five countries, including the United States, France, Italy, Spain, and Canada. PIV access was performed in the ED in 13 studies, in the ICU, and in the Anesthesia unit in one study. Moreover, 11 studies were RCTs, and the other four were observational (Table 1).

**TABLE 1:** Summary of study characteristics.

Author ID	Study design	Study location (study) setting	Participants				Venous visualization	No. of operators	Technique for US‐guidance	Reported outcomes
			Sample (*n*)	M/F	Age group	Study population				
(Bahl et al. 2016)	Prospective RCT	United States (ED)	122	32/90	Adults	Patients with a history of being a “difficult stick,” experienced at least two failed PIV access or had a history of end‐stage renal disease, IV drug abuse, and sickle cell disease.	NR	Single	Dynamic	Successful cannulation and procedure time.
(Bair et al. 2008)	RCT	United States (ED)	44	NR	Children	Patients requiring IV access and with a failed first IV attempt	NR	Dual	Static	First attempt success
(Bauman et al. 2009)	Prospective cohort study	France (ED)	75	21/54	Adults	Patients with at least two failed PIV access attempts	Transverse	Single	Dynamic	Successful cannulation, number of attempts, procedure time, and complications.
(Benkhadra et al. 2012)	Prospective RCT	France (Anesthesia unit)	40	27/13	Children	Patients with no visible/palpable veins in at least one limb	Transverse or longitudinal	NR	NR	Successful cannulation, number of attempts, and procedure time.
(Bridey et al. 2018)	Prospective RCT	France (ICU)	114	44/68	Adults	Patients awake in the ICU who no longer need CIVC and without apparent or palpable veins in the upper limbs due to BMI > 30, fluid overload, history of IV drug abuse, or chemotherapy.	NR	NR	NR	Successful cannulation, first attempt success, and number of attempts.
(Costantino et al. 2005)	Prospective RCT	United States (ED)	60	NR	Adults	Patients with at least three failed attempts or history of difficult access due to obesity, IV drug abuse, or chronic medical conditions	Transverse	Dual	Dynamic	Successful cannulation, procedure time, number of attempts, and complications.
(D'Alessandro et al. 2024)	Prospective observational cohort study	Italy (ED)	110	66/44	Children	Patients with a DIVA score ≥ 4 or a history of more than three attempts to attain the vein line or a previous venous line placed by anesthesiologists.	Transverse or longitudinal	Single	Dynamic	Successful cannulation, first attempt success, procedure time, number of attempts, and complications.
(Doniger et al. 2009)	Prospective RCT	United States (ED)	50	25/25	Children	Patients with at least two failed PIV access attempts or a history of difficult access	Transverse	Dual	Dynamic	Successful cannulation, procedure time, number of attempts, and complications.
(İsmailoğlu et al. 2015)	Descriptive study	Turkey (ED)	60	25/35	Adults	Patients not connected to a mechanical ventilator, did not need CIVC, not in critical condition, and with a history of difficult access due to obesity, peripheral oedema, dehydration, and chronic diseases.	Transverse	Dual or single	Dynamic	Successful cannulation, first attempt success, number of attempts, and complications.
(McCarthy et al. 2016)	RCT	United States (ED)	192	60/132	Adults	Patients with difficult PIV access.	NR	Single	Dynamic	First attempt success and complications
(Rodríguez‐Herrera et al. 2022)	Case–control study	Spain (ED)	72	28/44	Adults	Patients with non‐palpable or non‐visible veins	Transverse	NR	Dynamic	First attempt success and complications
(Stein et al. 2009)	Prospective RCT	United States (ED)	59	21/38	Adults	Patients with at least 2 failed PIV attempts	NR	Dual or single	Dynamic	Successful cannulation, number of attempts, procedure time, and complications.
(Vinograd et al. 2019)	Prospective RCT	(ED)	167	82/85	Children	Patients with a validated difficult intravenous access score ≥ 3	Transverse	Single	Dynamic	Successful cannulation, first attempt success, and complications
(Weiner et al. 2013)	Prospective RCT	United States (ED)	50	17/33	Adults	Patients with at least two failed PIV access attempts or a history of difficult access	Transverse	Single	Dynamic	Successful cannulation, procedure time, and number of attempts.
(Yalçınlı et al. 2022)	Prospective RCT	Turkey (ED)	270	103/167	Adults	Patients with no visible/palpable veins and those assessed to have difficult vascular access by a senior nurse	Transverse or longitudinal	Single	NR	First attempt success, procedure time, and number of attempts.

### 3.3 Successful IV Placement

**FIGURE 2:**
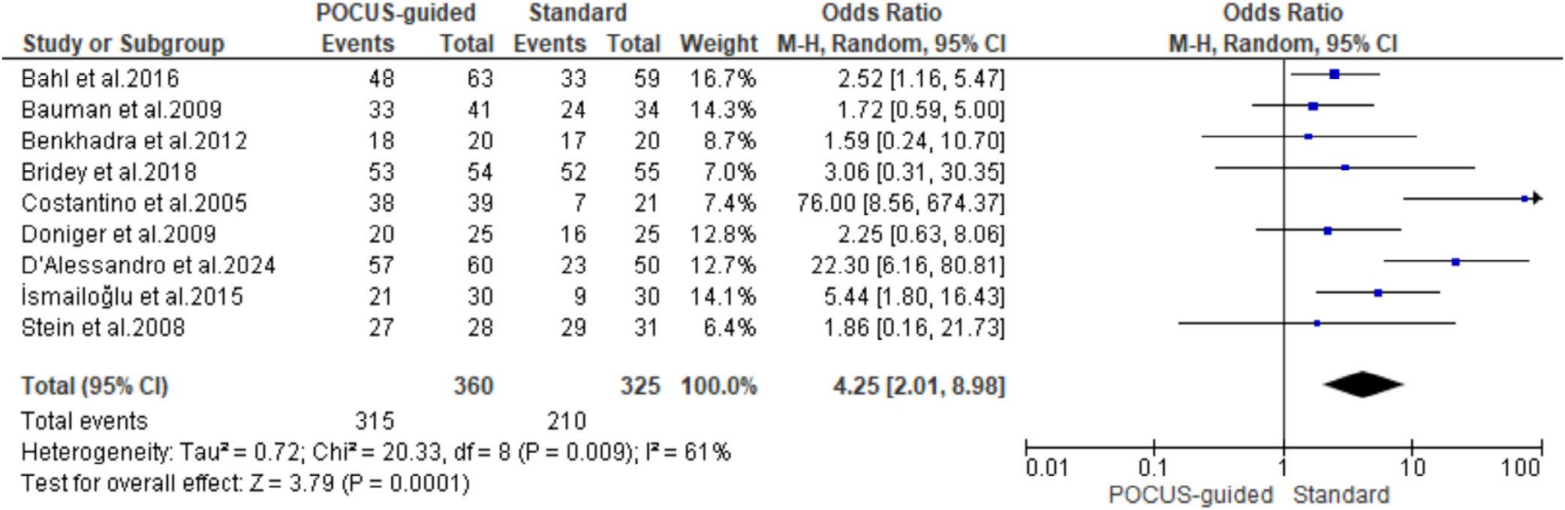
Forest plot comparing the rate of successful IV‐line placement.

Nine of the fifteen included studies reported the overall success rate for PIV access using the POCUS guidance. The pooled results have shown that POCUS guidance resulted in significantly higher rates of successful IV placement compared to the standard techniques (OR: 4.25; 95% CI: 2.01–8.98; *p* = 0.0001). However, these results demonstrated a high interstudy heterogeneity (*I* ^2^ = 61%) (Figure 2).

### 3.4 First‐Attempt Success

**FIGURE 3:**
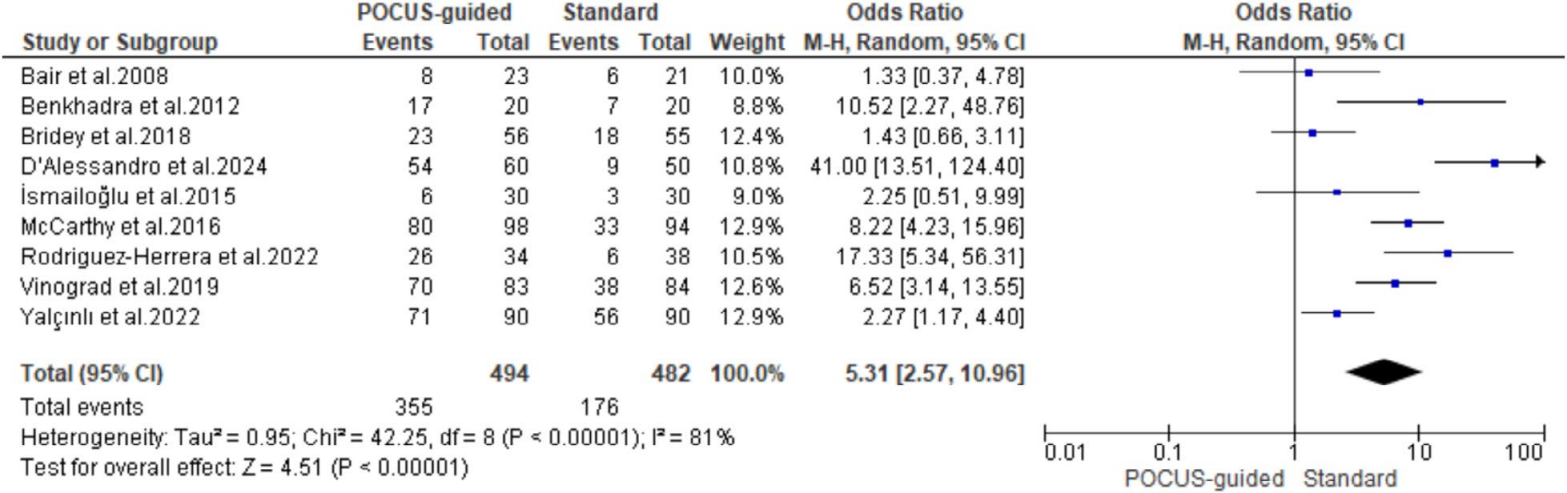
Forest plot comparing the rate of first‐attempt success.

Successful IV placement at the first attempt was reported in nine studies involving 966 patients with difficult venous access. Data pooled from these studies revealed that POCUS guidance significantly increased the first‐attempt success rate compared to standard techniques (OR: 5.31; 95% CI:2.57–10.96; *p* < 0.00001). However, we observed high interstudy heterogeneity in the pooled results (*I* ^2^ = 81%) (Figure 3).

### 3.5 Number of Attempts

**FIGURE 4:**
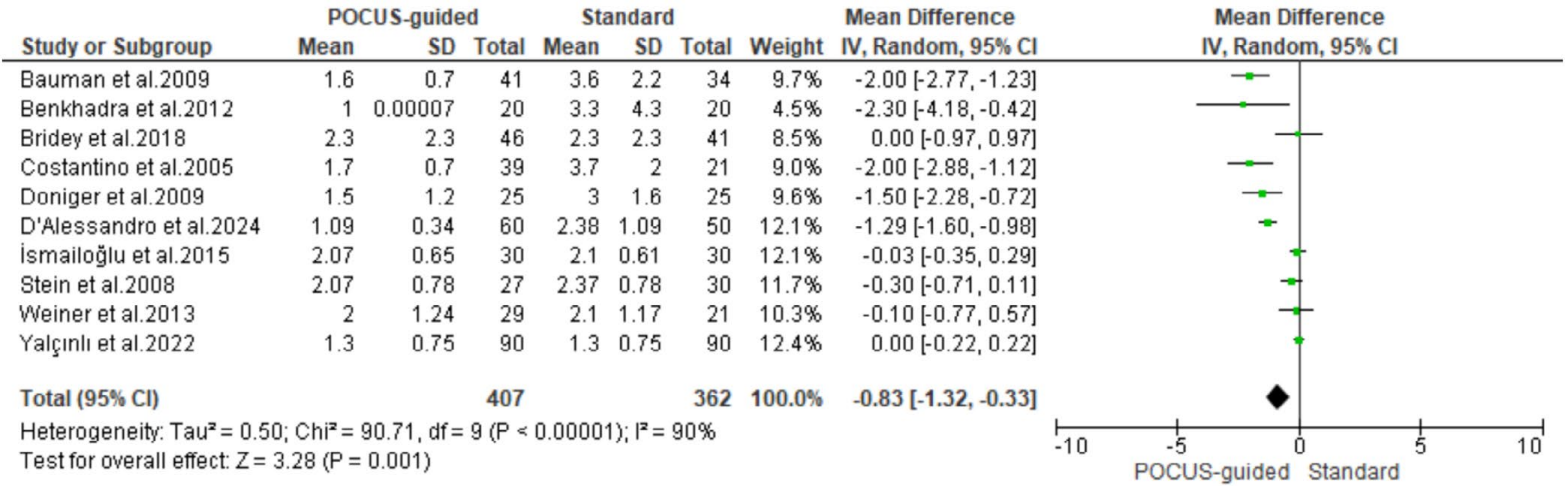
Forest plot comparing the mean number of attempts.

Ten studies involving 789 patients with difficult venous access provided sufficient data on the average number of attempts to achieve successful IV‐line placement. The pooled results showed that POCUS guidance resulted in significantly fewer skin puncture attempts than the standard technique (MD: −0.83; 95% CI: −1.32 – −0.33; *p* = 0.001). Nonetheless, there was evidence of interstudy heterogeneity (*I* ^2^ = 90%) (Figure 4).

### 3.6 Procedure Time

**FIGURE 5:**
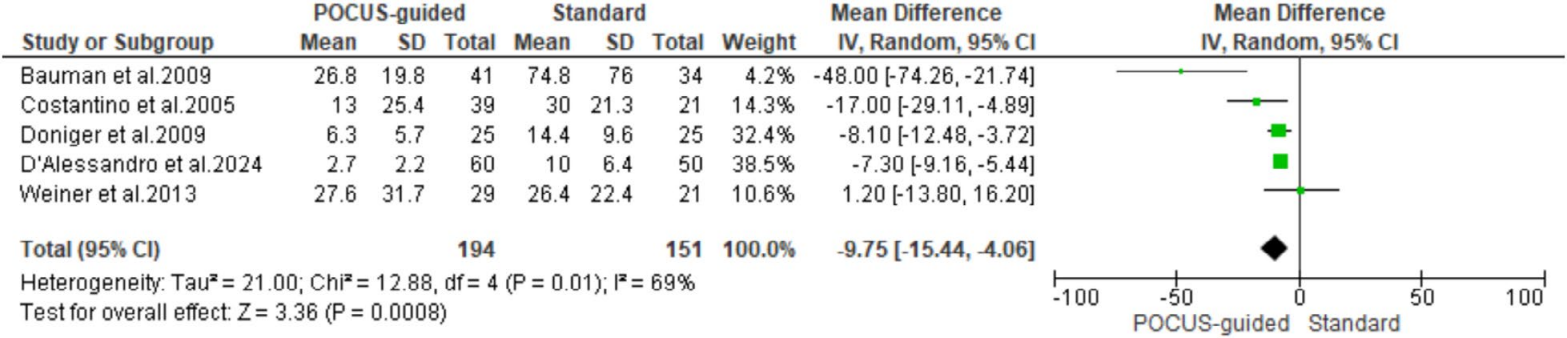
Forest plot comparing the procedure time.

Five studies reported the time taken to achieve successful IV‐line placement. The pooled analysis revealed that the POCUS guidance procedure time was significantly shorter than the standard technique (MD: −9.75 min; 95% CI: −15.44 – −4.06; *p* = 0.0008). This finding also showed a high heterogeneity across the studies (*I* ^2^ = 69%) (Figure 5).

### 3.7 Complications

**FIGURE 6:**
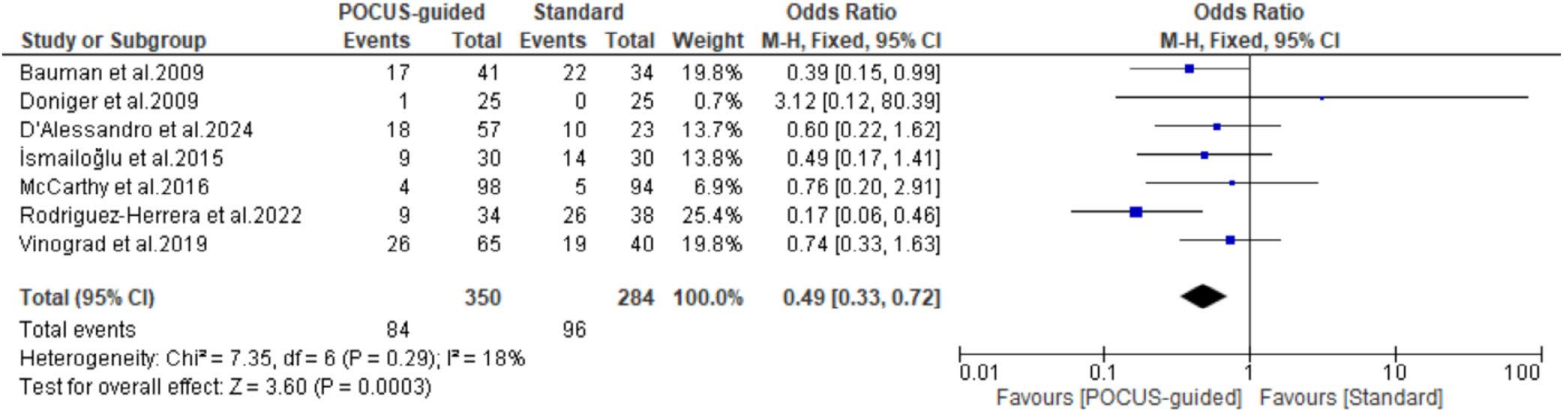
Forest plot comparing the number of complications.

**FIGURE 7:**
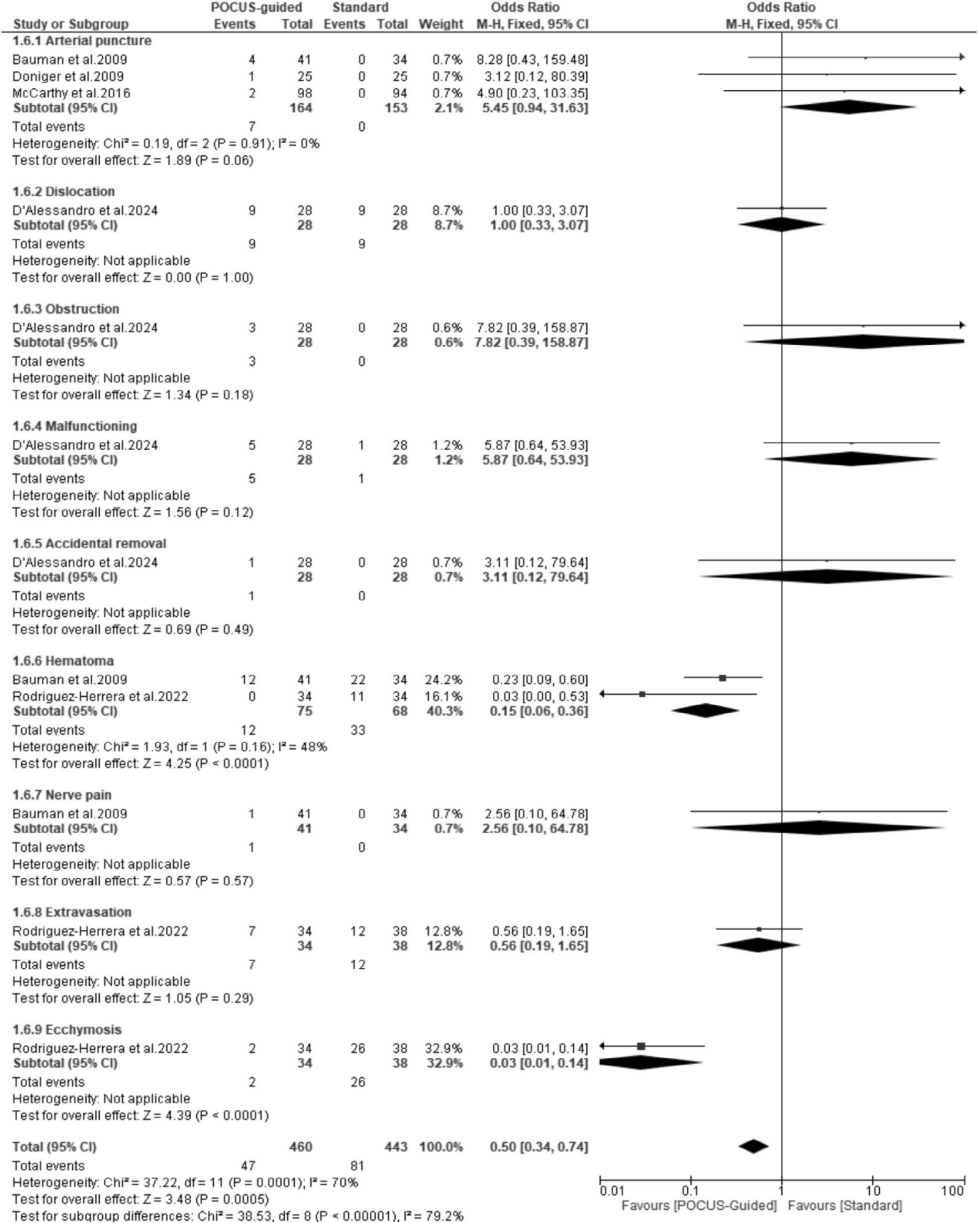
Forest plot comparing different types of complications.

Pooled results from seven studies revealed that the POCUS‐guided group had significantly fewer complications (84/350) compared to the standard technique group (96/284) (OR: 0.49; 95% CI: 0.33–0.72; *p* = 0.0003; *I* ^2^ = 18%) (Figure 6). A further investigation into the different types of complications revealed no significant difference between the two groups in the incidence of arterial puncture (*p* = 0.06), dislocation (*p* = 1.00), obstruction (*p* = 0.18), malfunctioning (*p* = 0.12), accidental removal (*p* = 0.49), nerve pain (*p* = 0.57), and extravasation (*p* = 0.29). However, the POCUS‐guided group showed significantly fewer incidences of hematoma (OR: 0.15; *p* < 0.0001) and ecchymosis (OR: 0.03; *p* < 0.0001) compared to the standard group (Figure 7).

### 3.8 Subgroup Analyses

The results on successful IV‐line placement, first‐attempt success, number of attempts, and procedure time demonstrated high interstudy heterogeneity; therefore, we performed subgroup analyses to identify the sources of heterogeneity. The analyses showed that neither the study design, setting, age group, venous visualization, number of operators, nor the technique for ultrasound guidance was a significant source of heterogeneity for successful IV placement. Therefore, the heterogeneity in this outcome was likely caused by other factors or a combination of the analyzed factors. On the other hand, the analyses showed that the study setting, the number of operators, and the technique for ultrasound guidance contributed to the heterogeneity in first‐attempt success. Our analysis also revealed that the number of operators contributed to the number of skin puncture attempts. At the same time, the heterogeneity in procedure time can be attributed to the study design (Table 2).

**TABLE 2:** Subgroup analyses to identify sources of heterogeneity.

Outcome	Covariates	Subgroups	No. of studies	Test for subgroup differences (*p*)
Successful IV placement	Study design	RCT	7	0.53
		Observational	2	
	Study setting	ED	7	0.56
		ICU	1	
		Anesthesia unit	1	
	Age group	Adults	6	0.86
		Children	3	
	Venous visualization	Transverse	4	0.07
		Transverse or longitudinal	2	
		NR	3	
	Number of operators	Single	3	0.75
		Dual	2	
		Dual or single	2	
		NR	2	
	Technique for ultrasound guidance	Dynamic	7	0.32
		NR	2	
First attempt success	Study design	RCT	7	0.30
		Observational	2	
	Study setting	ED	7	0.01
		ICU	1	
		Anesthesia unit	1	
	Age group	Children	4	0.39
		Adults	5	
	Venous visualization	Transverse	3	0.42
		Transverse or longitudinal	3	
		NR	3	
	Number of operators	Single	4	0.05
		Dual	1	
		Single of dual	1	
		NR	3	
	Technique for ultrasound	Static	1	< 0.00001
		Dynamic	5	
		NR	3	
Number of attempts	Study design	RCT	8	0.87
		Observational	2	
	Study setting	ED	8	0.08
		ICU	1	
		Anesthesia unit	1	
	Venous visualization	Transverse	5	0.16
		Transverse or longitudinal	2	
		NR	2	
	Number of operators	Single	4	< 0.0001
		Dual	2	
		Single or dual	2	
		NR	2	
	Technique for ultrasound guidance	Dynamic	7	0.25
		NR	3	
Procedure time	Study design	RCT	4	0.003
		Observational	1	
	Venous visualization	Transverse	4	0.31
		Transverse or longitudinal	1	
	Number of operators	Single	3	0.77
		Dual	2	

### 3.9 Quality Appraisal Outcomes

**FIGURE 8:**
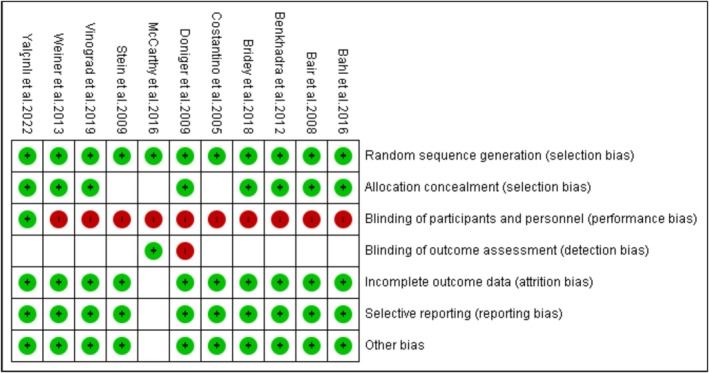
Risk of bias summary.

The risk of bias assessment has shown that all studies except one have a high risk of performance bias because patients were not blinded to the interventions. The evaluation also indicates that only one study has a high risk of bias because outcome assessors were not blinded (Figure 8). On the other hand, the assessment using NOS showed that all studies had fair methodological quality. Details of the NOS assessment are provided in Appendix B (available in the Supporting Information).

## 4 Discussion

PIV access is a routine procedure in many medical institutions. However, some patients often present with difficult PIV access, which requires them to undergo multiple attempts by several healthcare providers and sometimes may require central venous access, thus increasing procedure time and resource use. As such, POCUS has emerged as a valuable tool for guiding PIV access. Therefore, this systematic review and meta‐analysis investigated the efficacy and safety of POCUS‐guided PIV in patients with difficult intravenous access.

The present study found that POCUS guidance improved the overall success and first‐attempt success in obtaining PIV access. This finding compares favorably with four previous meta‐analyses, which found that the first‐attempt success and overall success rates were significantly higher with ultrasound guidance (Egan et al. 2013; Stolz et al. 2015; Álvarez‐Morales et al. 2024; Tran et al. 2021). Contrary to these findings, Bair and colleagues found no considerable difference in first‐attempt success between the ultrasound and standard technique groups (35% vs. 29%, respectively) (Bair et al. 2008). This difference can be attributed to the fact that, unlike other studies that revealed ultrasound to be useful for IV insertion, it employed the “static” ultrasound technique, which is an outdated ultrasound technique that has been replaced by the real‐time (dynamic) technique. Nonetheless, the authors of that study claimed that additional skin marks might have improved the cannulation success. Therefore, further research in large‐sample prospective RCTs is needed to ascertain whether the “static” ultrasound technique employing multiple skin marks might improve cannulation success rates.

This review also showed that POCUS guidance significantly reduced the number of skin puncture attempts and the time needed to conduct the procedure. Therefore, POCUS guidance is timesaving and can result in improved patient satisfaction. Indeed, Baumann and colleagues found that ultrasound guidance resulted in significantly higher patient satisfaction than the traditional technique among adult patients with difficult intravenous access (Bauman et al. 2009). Similar findings have also been reported in other studies (Costantino et al. 2005; Stein et al. 2009). Contrary to our finding, two previous meta‐analyses involving patients with difficult venous access found no significant difference in the number of skin puncture attempts and procedure time between ultrasound guidance and standard technique groups (Egan et al. 2013; Stolz et al. 2015) Although we cannot ascertain the reason for this difference, it might be associated with the fact that they included fewer studies than our meta‐analysis.

Interestingly, our subgroup analyses suggested that the number of operators involved in POCUS guidance might impact the success of PIV access at the first attempt. These analyses showed that the single‐operator technique improved first‐attempt success, but no difference was observed with the dual‐operator technique. Nonetheless, the evidence on the dual‐operator technique was based on the study by Bair et al. (2008), which also involved the “static” ultrasound technique. Therefore, the non‐significant difference in the first‐attempt success rate was likely related to the ultrasound technique rather than the number of operators. Indeed, Rose and colleagues found no considerable difference between the single‐operator and dual‐operator techniques for ultrasound‐guided cannulation of the basilic vein (Rose and Norbutas 2008). Considering these findings, we believe that one or more operators can effectively perform POCUS‐guided PIV access.

In addition, our meta‐analysis has shown that POCUS guidance results in significantly fewer complications compared to the standard technique. Therefore, POCUS‐guided PIV access can be considered a safe procedure for patients with difficult venous access. Nonetheless, our results have shown that although non‐significant, the incidences of arterial puncture were more common in the POCUS‐guided group than in the standard technique group (4.27% vs. 0%). This finding is not surprising as previous literature has shown that arterial puncture occurs in about 2% of the patients subjected to POCUS‐guided PIV access (Keyes et al. 1999; Brannam et al. 2004). Notably, Bauman et al. (2009) found a high number of arterial punctures in the ultrasound group compared to the standard group (10% vs. 0%). This high incidence of arterial puncture might have occurred because inexperienced ED technicians performed ultrasound‐guided PIV access. Indeed, the authors reported that one of the arterial punctures occurred because the ED technician incorrectly interpreted the blue Doppler signal as definitive for a vein, and another occurred after an ED technician attempted the vein on top artery configuration. Considering this finding, POCUS‐guided PIV access must be performed by experienced personnel or by personnel with sufficient training in ultrasound.

### 4.1 Limitations

The current meta‐analysis has some limitations that should be noted when interpreting its findings. First, we imposed language restrictions, meaning that non‐English records were not eligible for inclusion. This restriction might have introduced selection bias in our study. Second, we should have analyzed patient‐reported outcomes, such as patient satisfaction and pain rating scores, since they were only reported in a few studies. Therefore, future research should strive to report these outcomes as they are important for a patient‐oriented healthcare system. Finally, the definition of difficult venous access highly varied across the included studies, and this might have also contributed to significant heterogeneity in the pooled outcomes.

## 5 Conclusion

In summary, POCUS‐guided PIV access is a practical, time‐saving, and safe medical procedure for patients with difficult venous access. Therefore, we recommend the use of POCUS to guide PIV access among patients with difficult venous access or those with failed venous access using standard procedures. Nonetheless, evidence suggests that POCUS‐guided PIV access performed by inexperienced healthcare personnel might result in increased complications. Therefore, healthcare personnel, especially those in the ED, ICU, and anesthesia unit, should be subjected to more structured educational programs on POCUS‐guided PIV access to help them gain confidence and proficiency in administering this medical procedure.

## Conflicts of Interest

The authors declare no conflicts of interest.

## Supporting information


**Appendix S1.** Supporting Information.

## Data Availability

The data that support the findings of this study are available from the corresponding author upon reasonable request.
