# Supplementary material for: Outcomes of POCUS‐Guided Peripheral Intravenous Access in Difficult Venous Access Patients: A Systematic Review and Meta‐Analysis
Source: J Clin Ultrasound. 2025 May 7;53(8):1846–59. doi: 10.1002/jcu.24059 (PMC12497679; doi:10.1002/jcu.24059)
Supplement: Supplementary file 1 — Appendix S1. Supporting Information. [file JCU-53-1846-s001.docx]

**Appendix A:** Search Strategy

1. **PubMed:**

**[All Fields]** (point-of-care ultrasound OR bedside ultrasound OR portable ultrasound OR hand-held ultrasound OR POCUS OR ultrasound OR sonography) AND (peripheral intravenous access OR peripheral venous access OR peripheral access OR peripheral catheter OR peripheral intravenous line OR peripheral catheter placement) AND (difficult access OR difficult intravenous access OR difficult venous access OR difficult patients)

**[Title/Abstract]** (point-of-care ultrasound OR bedside ultrasound OR portable ultrasound OR hand-held ultrasound OR POCUS OR ultrasound OR sonography) AND (peripheral intravenous access OR peripheral venous access OR peripheral access OR peripheral catheter OR peripheral intravenous line OR peripheral catheter placement) AND (difficult access OR difficult intravenous access OR difficult venous access OR difficult patients)

1. **Cochrane Library:** (point-of-care ultrasound OR bedside ultrasound OR portable ultrasound OR hand-held ultrasound OR POCUS OR ultrasound OR sonography) AND (peripheral intravenous access OR peripheral venous access OR peripheral access OR peripheral catheter OR peripheral intravenous line OR peripheral catheter placement) AND (difficult access OR difficult intravenous access OR difficult venous access OR difficult patients)
2. **MEDLINE:** (point-of-care ultrasound OR bedside ultrasound OR portable ultrasound OR hand-held ultrasound OR POCUS OR ultrasound OR sonography) AND (peripheral intravenous access OR peripheral venous access OR peripheral access OR peripheral catheter OR peripheral intravenous line OR peripheral catheter placement) AND (difficult access OR difficult intravenous access OR difficult venous access OR difficult patients)
3. **Web of Science:** (point-of-care ultrasound OR bedside ultrasound OR portable ultrasound OR hand-held ultrasound OR POCUS OR ultrasound OR sonography) AND (peripheral intravenous access OR peripheral venous access OR peripheral access OR peripheral catheter OR peripheral intravenous line OR peripheral catheter placement) AND (difficult access OR difficult intravenous access OR difficult venous access OR difficult patients)
4. **Google Scholar:**

**[With all of the words]** (point-of-care ultrasound OR bedside ultrasound OR ultrasound) AND (peripheral intravenous access) AND (Difficult venous access)

**[With at least one of the words]** (point-of-care ultrasound OR bedside ultrasound OR portable ultrasound OR hand-held ultrasound OR POCUS OR ultrasound OR sonography) AND (peripheral intravenous access OR peripheral venous access OR peripheral access OR peripheral catheter OR peripheral intravenous line OR peripheral catheter placement) AND (difficult access OR difficult intravenous access OR difficult venous access OR difficult patients)

**Appendix B: Assessment of Methodological Quality of Included Studies Using the Newcastle-Ottawa Scale (NOS)**

| Study ID | Exposed cohort representativeness | Non-exposed cohort selection | Exposure verification | Initial outcome absence | Cohort comparability (Design/Analysis adjusted for confounders) | Outcome evaluation | Sufficient follow-up | Cohort follow-up | Total score | Overall quality |
| --- | --- | --- | --- | --- | --- | --- | --- | --- | --- | --- |
| Bauman et al.2009 | 0 | 1 | 1 | 1 | 0 | 0 | 1 | 1 | 5 | Fair |
| D’Alessandro et al.2024 | 0 | 1 | 1 | 1 | 0 | 0 | 1 | 1 | 5 | Fair |
| İsmailoğlu et al.2015 | 0 | 1 | 1 | 1 | 0 | 0 | 1 | 1 | 5 | Fair |
| Rodriguez-Herrera et al.2022 | 0 | 1 | 1 | 1 | 0 | 0 | 1 | 1 | 5 | Fair |
